# Surgical Correction of Large Talar Tilt in Varus Ankle Osteoarthritis: Lessons from Clinical Experience and a Review of the Literature

**DOI:** 10.3390/jcm14082781

**Published:** 2025-04-17

**Authors:** Jun Young Choi, Jin Soo Suh

**Affiliations:** Department of Orthopedic Surgery, Inje University Ilsan Paik Hospital, 170 Juhwa-ro, Ilsanseo-gu, Goyang 10380, Gyeonggi-do, Republic of Korea; sjs0506@paik.ac.kr

**Keywords:** ankle, osteoarthritis, varus talar tilt, joint-preserving surgery, supramalleolar osteotomy, inframalleolar correction

## Abstract

Numerous studies exist on medial opening wedge supramalleolar osteotomy (SMO), ever since its introduction by Takakura et al., as a joint-preserving surgical option for treating varus ankle osteoarthritis (OA). Although SMO can induce lateral translation of the talus—which is medially translated in varus ankle OA—it has only minimal effects on the correction of the varus tilt of the talus. Particularly, SMO alone does not effectively neutralize the talar position. The primary reason for this limitation is that varus tilting of the talus is not merely a two-dimensional deformity in the coronal plane, but rather a three-dimensional deformity involving internal rotation and anterior subluxation. Therefore, this study aimed to explore the key considerations for achieving effective correction of varus talar tilt in joint-preserving surgery for treating degenerative varus ankle OA with large talar tilting. Further, we have discussed the relevant studies and included the lessons learned from our clinical experience, categorizing the key surgical considerations into preoperative, intraoperative, and postoperative phases.

## 1. Introduction

Biomechanically, the ankle joint—a highly congruent joint—bears four times the body weight in a standing position [1]. While it has a relatively small contact area—approximately 33% of that of the hip or knee joint [2]—its cartilage thickness is also thinner than that of the other joints [3]. However, the ankle joint is structurally more resistant to shear and tensile forces, which helps reduce the joint contact pressure [3]. This characteristic contributes to the fact that symptomatic osteoarthritis (OA) of the ankle joint is nine times less frequent than that of the knee joint [3,4]. Nevertheless, when the balance between the tibial plafond and talus is disrupted, and varus or valgus tilting forces are persistently applied to the talus, alterations in the joint contact pressure distribution occur. This, in turn, leads to asymmetric cartilage wear, ultimately progressing to ankle OA.

Ankle OA affects approximately 1% of the adult population [5,6], with degenerative OA being relatively rare [1,7,8]. Most cases are associated with prior trauma around the ankle [7]. In the absence of preceding trauma, secondary causes such as rheumatoid arthritis or changes in knee joint alignment are often considered. Ankle OA can be broadly categorized into the valgus and varus types. Valgus ankle OA is commonly associated with post-traumatic changes or progressive collapsing foot deformity [9,10]. In contrast, varus ankle OA can arise from post-traumatic changes, and also has a definitive presence as a degenerative condition. Although the exact incidence of degenerative varus ankle OA remains unclear, several related studies have been published in East Asia compared with Western societies, suggesting potential ethnic differences in its prevalence.

Since Takakura et al. [11] introduced medial opening wedge supramalleolar osteotomy (SMO) as a joint-preserving surgical option for varus ankle OA, numerous follow-up studies have been published. Recent systematic or narrative reviews [12,13,14,15,16] have consistently reached a general consensus that while SMO can induce lateral translation of the talus—which is medially translated in varus ankle OA—it has only limited effects in correcting the varus tilt of the talus. In particular, SMO alone does not effectively neutralize (valgus-rotate) the talar position. The primary reason for this limitation is that varus tilting of the talus is not merely a two-dimensional deformity in the coronal plane but rather a three-dimensional deformity involving internal rotation and anterior subluxation [17]. While some reports claim that SMO alone can correct talar internal rotation [18], our clinical experience suggests that correction performed exclusively in the coronal plane results in highly unpredictable changes in talar tilting.

Kurokawa et al. [19] reported that talar tilt is the most significant risk factor for the progression of varus ankle OA and that a varus talar tilt greater than 2° increases the risk of OA progression, making its correction crucial. Therefore, this study aimed to explore the key considerations for achieving effective correction of varus talar tilt in joint-preserving surgery for degenerative varus ankle OA with large talar tilting. We have discussed the relevant studies and the lessons learned from our clinical experience, categorizing the key surgical considerations into preoperative, intraoperative, and postoperative phases.

## 2. Preoperative Considerations

### 2.1. Fundamental Radiographic Parameters

**Figure 1:**
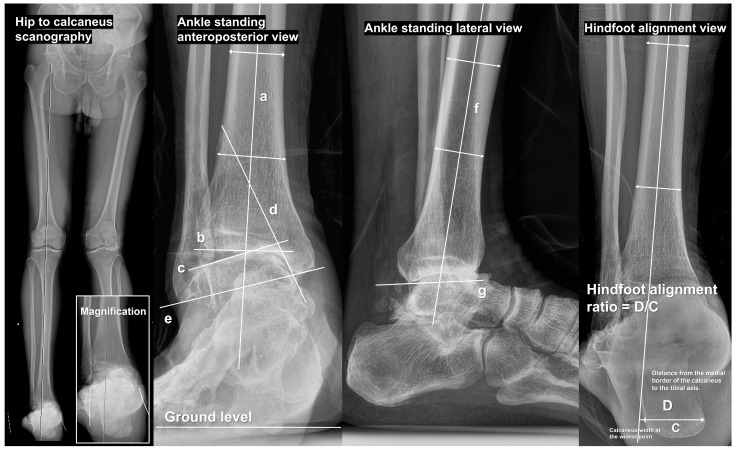
Schematic measurements of radiographic parameters. On hip-to-talus scanography, a line connecting the center of the femoral head and ankle joint is drawn to assess the position of the knee joint along this line to evaluate knee joint alignment. This line can be extended distally to evaluate its relationship with the hindfeet. In the ankle standing anteroposterior view, the tibial anterior surface angle is defined as the medial angle between (a) the tibial axis and (b) tibial plafond. Furthermore, the talar tilt (between (b) and (c)), tibiomedial malleolar angle (between (a) and (d)), and talocrural angle (between (b) and (e)) should be measured. The tibial plafond inclination (between ground level and (b)) determines the position of the tibial plafond on the ground. The tibial lateral surface angle is the angle between (f) and (g) in the ankle standing lateral view. We evaluated the hindfoot moment arm and hindfoot alignment ratio in the hindfoot alignment view. The former is defined as the distance between the lowest point of the calcaneus and tibial axis, whereas the latter is the distance from the medial border of the calcaneus to the tibial axis (D) divided by the width of the calcaneus at its widest point (C). In most cases of varus ankle osteoarthritis, excessive varus malalignment of the hindfoot results in a calculated value greater than 1.

In radiographical evaluation of the degenerative varus ankle OA, lower-extremity scanography to assess the overall alignment of the lower limbs is of paramount importance. Whole lower-extremity images can be obtained using hip-to-talus and hip-to-calcaneus methods [20]. Among these, hip-to-calcaneus scanography is recommended as it allows the evaluation of the entire lower extremity alignment, including hindfoot alignment. In this image (Figure 1), a line connecting the center of the femoral head and ankle joint can be drawn to assess the position of the knee joint along this line, thereby evaluating knee joint alignment. If knee joint malalignment is present, its correction should be prioritized. Knee joint malalignment was not observed in many patients with degenerative varus ankle OA. In such cases, the line connecting the femoral head and ankle joint center is extended distally to evaluate its relationship with the hindfoot. In most cases, the hindfoot exhibits varus malalignment relative to this line.

Several radiographical parameters measurable on the ankle standing anteroposterior (AP) and lateral views have been introduced and analyzed in previous studies. We have summarized only the most fundamental parameters and those that were critically assessed preoperatively in Table 1, and Figure 1 illustrates how each parameter was measured.

In addition to these parameters, standing AP, lateral, and internal/external oblique views of the foot can be obtained to assess the presence of cavus foot deformity and concomitant foot arthritis. Recently, weight-bearing computed tomography (WBCT) has been increasingly utilized for more precise evaluation of deformity characteristics. WBCT has the advantage of assessing whether the hindfoot compensates for the varus deforming forces of the ankle joint and provides a three-dimensional visualization of the talus position relative to the tibial plafond [21,22,23,24,25].

### 2.2. Definition of Large Varus Talar Tilt and Surgical Limits

Joint-preserving surgery through SMO should be attempted with caution when there is a large varus talar tilt. Tanaka [26] suggested that a talar tilt exceeding 10° precludes postoperative attainment of a normal joint space. Lee et al. [27] proposed an optimal threshold of 7.3° for varus talar tilt, whereas Choi et al. [28] defined a talar tilt greater than 8° as a large varus talar tilt. A recent systematic review aimed at identifying the optimal degree of talar tilt correction [15] failed to reach a definitive conclusion. Based on our clinical experience, a talar tilt of up to 10° can be effectively managed with SMO and fibular-shortening osteotomy, yielding favorable outcomes. Cases with a talar tilt of up to 15° are also amenable to SMO; however, adjunctive inframalleolar correction (IMC) is necessary. In contrast, for varus talar tilts exceeding 15°, even when additional surgical techniques are employed, postoperative correction of the talar tilt remains unpredictable, with a substantial number of cases exhibiting a persistent deformity. Finally, in patients with a varus talar tilt of 20° or more, joint-sacrificing procedures, such as ankle arthrodesis or total ankle replacement arthroplasty, should be considered over joint-preserving surgery.

### 2.3. Type of Varus Ankle Osteoarthritis

**Figure 2:**
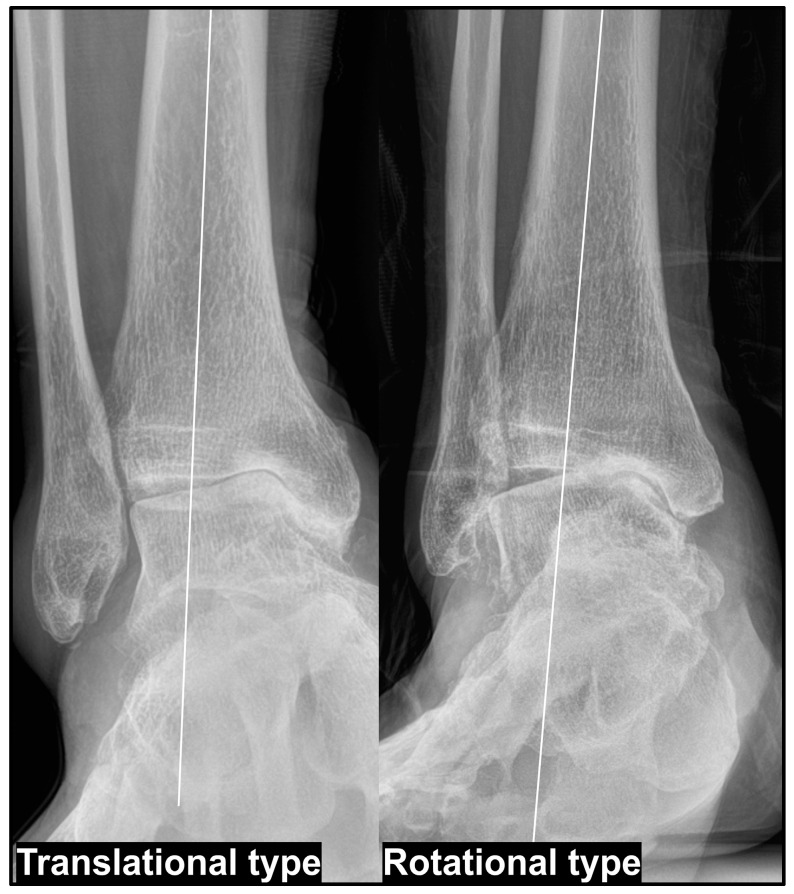
Two types of varus ankle osteoarthritis. The translational type develops as the traditional varus ankle osteoarthritis progresses, whereas the rotational type is mostly accompanied by the progression of pes cavovarus deformities. In the translational type, the medial talar gutter is in direct contact with the medial malleolus.

Takakura et al. [11] classified varus ankle OA into four stages: Stage I—early-stage arthritis with subchondral sclerosis and minimal joint space narrowing; Stage II—medial joint space narrowing without complete obliteration; Stage IIIA—obliteration of the joint space with subchondral bone contact between the medial talar gutter and medial malleolus; Stage IIIB—further progression of joint space obliteration, extending to the roof of the talar dome with subchondral bone contact; Stage IV—complete joint destruction with widespread osteoarthritis affecting both the medial and lateral compartments. According to this classification, as the disease progresses from Stage IIIA to Stage IIIB, erosion of the medial malleolus occurs, accompanied by an increase in varus talar tilt, leading to subchondral bone contact between the medial talar dome and the tibial plafond. Based on previous research [29], we propose to define this progressively occurring varus talar tilt—which develops as part of the natural course of degenerative varus ankle OA—as the “translational type.” In contrast, severe pes cavovarus deformity contributes to the development of a varus talar tilt, ultimately resulting in subchondral bone contact between the medial talar dome and the plafond. However, in such cases, the talus does not translate medially but remains relatively centered within the ankle joint. We define this pattern as the “rotational type” (Figure 2).

Based on our clinical experience, the degree of varus talar tilt tends to be more pronounced in the rotational type than in the translational type. Additionally, correction through SMO alone is often insufficient in the rotational type, wherein addressing the underlying pes cavovarus deformity plays a crucial role in treatment. In contrast, in the translational type, SMO effectively facilitates lateralization of the talus, and varus talar tilt can often be successfully corrected using additional procedures. Furthermore, in cases of the rotational type, it is essential to investigate the underlying cause of pes cavovarus and confirm whether a paralytic component is present using electromyography and nerve conduction velocity studies before surgery.

### 2.4. Radiographic Correction Goals

Traditionally, overcorrection of the tibial anterior surface angle (TAS) has been emphasized in SMO [12,14]. Tanaka et al. [30] and Lee et al. [27] reported successful outcomes with overcorrection targeting a TAS > 95°; however, a recent study by Choi et al. [31] argued that overcorrection of the tibial plafond to valgus does not seem necessary, and most studies indicate that the mean postoperative TAS is typically < 95°. Our clinical experience aligns with that of previous studies, suggesting that targeting a postoperative TAS between 92° and 94° is appropriate, with corrections exceeding 95° warranting caution.

**Figure 3:**
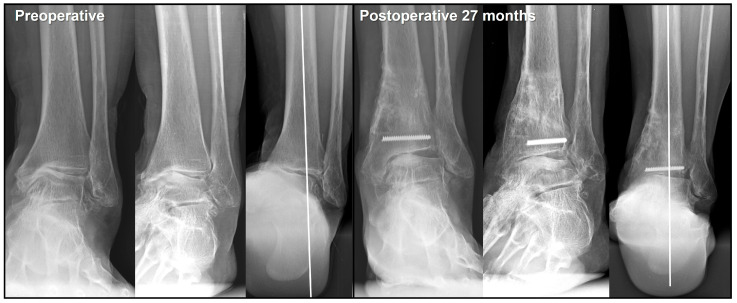
Paradoxical increase in talar tilt after supramalleolar osteotomy. Overcorrection of the tibial anterior surface angle (TAS) may paradoxically increase talar tilt.

Furthermore, we advocate the importance of considering tibial plafond inclination (TPI) when determining the degree of medial opening. The TPI represents the varus/valgus orientation of the tibial plafond relative to the ground level. Based on our experience, even when SMO and IMC are performed for varus ankle OA, the varus-tilted talus can be brought as close as possible to a neutral position relative to the ground, but does not shift into a valgus position. If the talus remains in a varus position to the ground postoperatively, excessive valgus positioning of the tibial plafond relative to the ground may paradoxically increase talar tilt, leading to rapid progression or aggravation of OA after surgery (Figure 3). Therefore, we recommend evaluating preoperative TPI and adjusting TAS to achieve a neutral or slightly valgus TPI, ideally remaining within 2 to 3° of valgus [32]. Based on these findings, it is recommended to determine the extent of medial opening accordingly. Assuming that each 1 mm medial opening results in approximately a 1°-correction of the TAS, this can serve as a practical guideline.

### 2.5. Age Limitation in Varus Talar Tilt Correction

Choi et al. [33] previously reported that there was no difference in the outcomes of SMO between patients aged ≥65 years and those aged <65 years. However, similar to joint-preserving surgery of the knee joint, SMO is generally not recommended for patients aged >70 years [4,9]. Nevertheless, with advancements in medical technology, it is now possible to expect bone union rates to be comparable to those in younger patients, even in individuals aged >70 years, and many older patients remain highly active. Therefore, SMO should be considered as a viable option for appropriately selected patients over the age of 70 years. Based on our clinical experience, we believe that the age limitation can be extended up to 75 years; however, this decision should be made with careful consideration of the patient’s nutritional status, medical history, and social support system.

## 3. Intraoperative Considerations: Supramalleolar Osteotomy

### 3.1. Osteotomy Type and Location

SMO for varus ankle OA can be broadly classified into medial opening wedge, lateral closing wedge [34], and dome osteotomy [35] based on its configuration. However, this study focused exclusively on medial opening wedge SMO. Medial opening wedge SMO can further be classified into conventional SMO, mortiseplasty [36,37,38,39], and plafond-plasty [40,41,42] based on the direction of the lateral end of the osteotomy line. In conventional SMO, the lateral end of the tibial osteotomy typically extends to the most proximal aspect of the syndesmosis [43]. In mortiseplasty, the osteotomy line is positioned slightly lower, directed toward the intrasyndesmotic area, without performing a fibular osteotomy. This approach has the advantage of reducing the ankle mortise gap in patients with varus ankle OA and concomitant medial malleolar erosion. The reason for omitting fibular osteotomy in mortiseplasty is based on the theoretical background that the fibula provides buttressing and stability during the valgization of the distal tibial fragment. Plafond-plasty directs the lateral end of the osteotomy line toward the ankle joint, thereby positioning the valgization correctional force directly at the center of rotation of angulation (CORA). However, hinge fractures may lead to iatrogenic damage to the plafond cartilage. Choi et al. [44] argued that distal syndesmotic SMO is advantageous in cases of ankle OA with a large varus talar tilt because it allows the deformity correction force to be applied closer to the CORA compared with conventional SMO.

Among the various osteotomy methods, we currently perform conventional SMO for varus ankle OA cases without a large varus talar tilt, but with medial translation of the talus. However, when correction of varus talar tilt is necessary, we perform distal syndesmotic SMO, in which the lateral end of the osteotomy is directed approximately 1–1.5 cm proximal to the ankle joint. The key difference from mortiseplasty is that in both cases, fibular osteotomy is always performed.

### 3.2. Fibular Osteotomy

Several studies have reported that fibular osteotomy during SMO contributes to ankle alignment correction [32,45,46,47,48]. The rationale for this is that the fibula plays a dual role in supporting the lateral joint and participating in load transmission [49,50,51,52,53]. The absence of this modest yet crucial weight-bearing function places increased pressure on the lateral plafond [50,53]. This is further illustrated by the loss of the buttressing function of the lateral malleolus anchored to the distal tibia. Although the number of related studies remains limited, preventing a concrete conclusion, we believe that fibular shortening plays a significant role in correcting varus talar tilt. Furthermore, the value and role of postoperative syndesmosis widening, as recently reported in the literature [32], warrant further attention. In cases of syndesmosis disruption, the talus may rotate into valgus or translate laterally, even when the deltoid ligament remains intact [54]. This effect can also be observed in post-traumatic or postoperative valgus talar tilt in patients with ankle fractures, even when only minimal displacement, shortening, or rotational deformity of the fibula is present [49,51,52]. If this concept can be reversed and applied to the correction of varus talar tilt, it may provide a key solution to this challenging issue [32].

**Figure 4:**
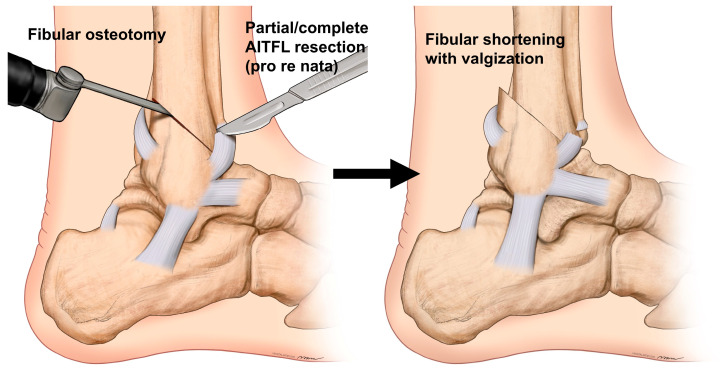
Fibular osteotomy for shortening and valgization. Fibular osteotomy is initiated near the attachment site of the anteroinferior tibiofibular ligament (AITFL) and extended posterosuperiorly obliquely.

Therefore, we currently perform fibular osteotomy in all patients who require varus talar tilt correction. In most cases, a huge bony spur forms at the anterior aspect of the lateral malleolus due to impingement between the talus and fibula. Therefore, its removal should be performed as the initial step [55]. We previously attempted various osteotomy configurations to achieve simultaneous shortening and valgization. However, we now perform the osteotomy in an oblique fashion [56], similar to the pattern observed in supination–external rotation-type ankle fractures, starting near the attachment site of the anteroinferior tibiofibular ligament (AITFL) on the anterior side and extending posterosuperiorly (Figure 4). The fibular osteotomy site was fixed after fixation of the tibial osteotomy site and IMC. Under fluoroscopic guidance, the ankle was positioned in maximum valgus and external rotation, and two 1.6 mm Kirschner wires were placed across the osteotomy site for stabilization. The advantage of this osteotomy is that it allows the distal fragment to naturally shorten and translate posteriorly along the osteotomy plane, while also permitting valgization, as needed. Additionally, the relatively broad osteotomy surface facilitates reliable union even with simple Kirschner wire fixation. To optimize the positioning of the distal fibular fragment, we sometimes perform a partial or total cut of the AITFL, when necessary [56].

### 3.3. Bone Marrow Stimulation of the Medial Talar Dome

**Figure 5:**
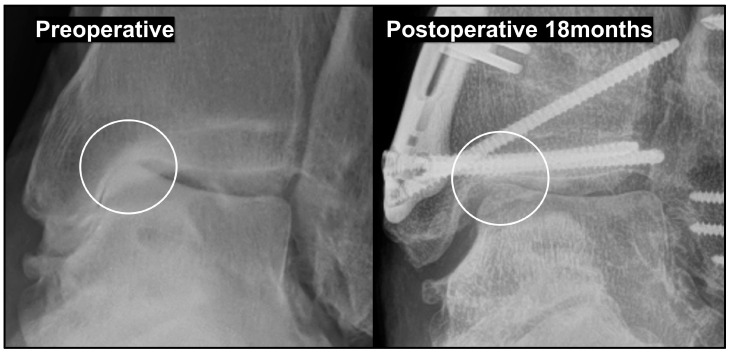
Postoperative gap formation between the medial talar dome and the plafond. The formation of a radiographic gap between the medial talar dome and the plafond is presumed to be directly associated with postoperative symptom improvement by eliminating subchondral bone contact.

Several studies have reported improvements in clinical outcomes, even without complete correction of the talar tilt, after joint-preserving surgery in patients with a large varus talar tilt. Based on our clinical experience, patients showed symptomatic relief as long as a radiographic gap was created between the medial talar dome and the plafond, even if the talar tilt was not fully corrected (Figure 5). This can be explained by the fact that simply shifting the talus laterally prevents subchondral bone contact between the talus and the plafond, and repositions the cartilage-denuded surface of the medial talar dome to an area where it contacts a cartilage-covered point of the plafond.

Additionally, promoting the formation of fibrous cartilage on the medial talar dome is important for maintaining the created gap. To achieve this, the incorporation of a bone marrow stimulation procedure during joint-preserving surgery should be considered [57]. Options for joint-preserving procedures include arthroscopic microfracture and open multiple drilling procedures. Lim et al. [58] reported that subchondral bone marrow stimulation was not necessary because fibrocartilage formation was observed on second-look arthroscopy, even after SMO alone. However, of the 29 patients included in their study, 26 had Takakura stage II or IIIA disease without a large varus talar tilt. Therefore, it is premature to conclude that bone marrow stimulation is unnecessary in patients with a large varus talar tilt. Consequently, we recommend incorporating a bone marrow stimulation procedure when performing joint-preserving surgery in patients with a large varus talar tilt.

### 3.4. Effects and Prevention of Hinge Fracture

Kim et al. [59] identified the presence of a lateral hinge fracture as a prognostic factor after SMO in patients with varus ankle OA. Park et al. [60] reported that postoperative computed tomographic evaluation revealed the presence of a lateral hinge fracture in 61.8% of cases following SMO. Some studies suggest that compared with high tibial osteotomy, SMO is more susceptible to lateral hinge fractures [60,61]. However, as no prior studies have established a correlation between the degree of medial opening and the occurrence of lateral hinge fractures, it is difficult to draw definitive conclusions. Based on our experience, when the medial opening exceeds 5 mm, careful attention is required to prevent lateral hinge fractures, and in most cases where the medial opening exceeds 7.5 mm, lateral hinge fractures are inevitable.

**Figure 6:**
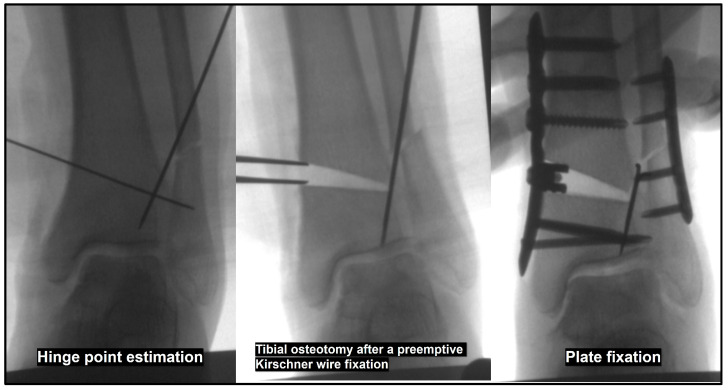
Preemptive Kirschner wire fixation for preventing lateral hinge fracture. Preemptive insertion of Kirschner wires at the anticipated hinge site along the virtual osteotomy line before tibial osteotomy can minimize the impact of hinge fractures.

A hinge fracture can lead to osteotomy site distraction during valgization of the distal tibial fragment, potentially resulting in osteotomy nonunion or lack of correction. To minimize the risk of hinge fracture, we recommend performing fibular osteotomy prior to tibial osteotomy. Also, another method to prevent hinge fractures or mitigate their effects if they occur is the preemptive fixation of 1.4 mm Kirschner wires at the anticipated hinge site before performing the tibial osteotomy (Figure 6) [61].

## 4. Intraoperative Considerations: Inframalleolar Correction

### 4.1. Calcaneal Osteotomy

Although SMO can play a role in ankle joint correction, IMC should be the primary treatment choice in cases in which varus talar tilt is a secondary change due to pes cavovarus deformity [62]. Moreover, even in cases where a large varus talar tilt occurs without a pes cavovarus component, supramalleolar correction has limited power to correct the talar tilt, making additional IMC essential [28]. Valgization calcaneal osteotomy plays a crucial role in directly altering hindfoot alignment [63] and includes lateral closing wedge osteotomy (Dwyer’s osteotomy [64]), lateral displacement osteotomy, and Z-shaped (rotational) osteotomy [65], with a frequently utilized option being a combination known as lateral displaced closing wedge osteotomy. Hintermann et al. [9,12] recognized the necessity of additional procedures beyond SMO for correcting varus talar tilt, but noted that the correction power of calcaneal osteotomy alone may be limited. Fundamentally, we agreed with this assertion and considered the degree of talar tilt correction achieved solely through calcaneal osteotomy to be unpredictable. However, when calcaneal osteotomy was combined with other IMC procedures, such as soft tissue procedures, the expected correction effect was found to be more reliable. Given that no definitive method for correcting varus talar tilt has been fully established, we advocate its inclusion as part of an all-in-one procedure along with SMO and shortening/valgization fibular osteotomy.

### 4.2. Soft Tissue Procedure on the Medial Aspect

Two key structures must be considered when performing soft tissue procedures on the medial aspect: the deltoid ligament and posterior tibial tendon (PTT). The deltoid ligament can be released from the medial malleolus to improve varus talar tilt, with two possible approaches: releasing only the superficial deltoid ligament [12] or releasing both the superficial and deep deltoid ligaments [66]. We prefer the approach of releasing both the superficial and deep deltoid ligaments.

**Figure 7:**
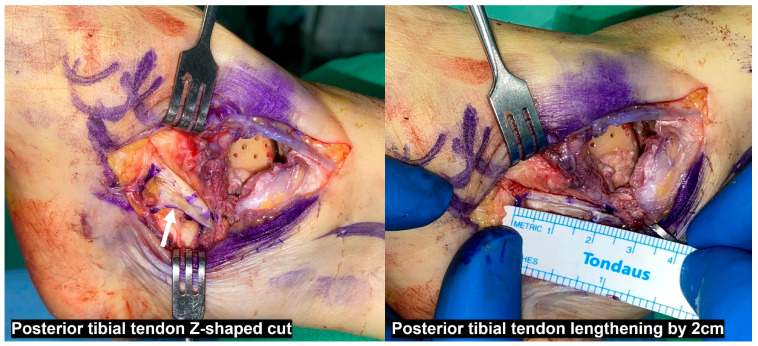
Posterior tibial tendon lengthening distal to the tarsal tunnel. A maximum lengthening of up to 2 cm is recommended.

As a powerful ankle plantar flexor and invertor, the PTT plays a crucial role in foot mechanics [67]. PTT transfer is known as a procedure used to restore ankle dorsiflexion in patients with peroneal nerve palsy. However, Park et al. [68] performed PTT transfer in 23 patients with nonparalytic ankle arthritis with large talar tilts to correct the deformity and reported favorable outcomes in 19 patients. However, because the PTT is a key structure in maintaining the medial longitudinal arch of the foot, its loss of function may lead to adverse effects, such as pes planovalgus. Therefore, careful consideration is required prior to performing this procedure. To mitigate these risks, PTT lengthening through a Z-shaped incision should be considered [56,69]. Our clinical experience suggests that distal lengthening performed beyond the tarsal tunnel is more effective. This is based on the anatomical observation that, within the tarsal tunnel, the PTT is relatively fixed by its sheath, limiting the efficacy of proximal lengthening in reducing the talus varus deforming force. Additionally, the PTT has a short tendon excursion distance of approximately 2 cm [67], indicating that its ability to stretch or lengthen is limited. Therefore, when performing PTT lengthening, a maximum of 2 cm is recommended (Figure 7).

### 4.3. Soft Tissue Procedure on the Lateral Aspect

Lateral soft tissue structure augmentation is as essential as the release or lengthening of medial soft tissue structures to improve varus talar tilt [12,63,69,70]. It primarily involves the anterior talofibular ligament (ATFL) and calcaneofibular ligaments (CFL). Preoperative evaluation of instability using stress radiographs and assessment of ligament integrity using ultrasonography or magnetic resonance imaging are crucial. Depending on the quality of the remnant ligament, treatment options include simple repair, augmentation with suture tape, or reconstruction using autograft or allograft transplantation.

### 4.4. Additional Procedures

In addition to the previously introduced procedures, other potential options include peroneus longus to brevis transfer, Achilles tendon lengthening, first metatarsal dorsal closing wedge osteotomy to correct forefoot pronation, and midtarsal osteotomy or subtalar/triple arthrodesis [71] for severe cavovarus deformities. Each of these procedures is important and should be considered based on the presence of a fixed deformity and any associated arthritis.

## 5. Surgical Sequence

**Figure 8:**
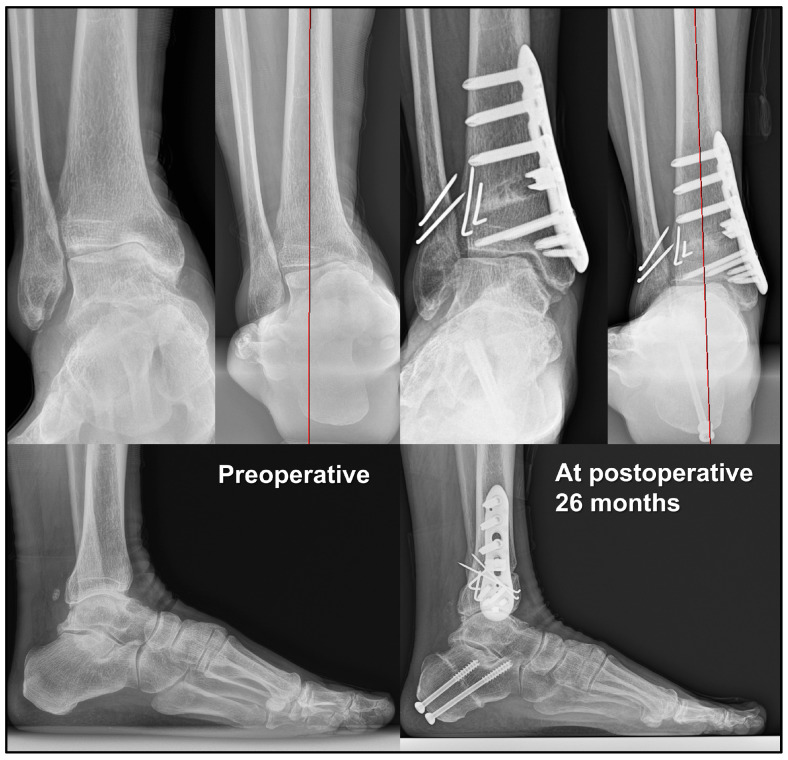
Corrected varus ankle osteoarthritis after the all-in-one procedure. Supramalleolar osteotomy, shortening/valgization fibular osteotomy with anteroinferior tibio-fibular ligament resection, lateral displaced closing wedge calcaneal osteotomy, deltoid ligament release, posterior tibial tendon lengthening, and anterior talofibular ligament repair augmented with suture tape are performed to salvage degenerative varus ankle osteoarthritis with a 12° talar tilt.

Currently, we perform an all-in-one procedure combining the previously introduced techniques for treating degenerative varus ankle OA with a large talar tilt (Figure 8). The surgical sequence is as follows:

Lateral position

1. Calcaneal osteotomy and internal fixation

Position change to supine

1. Fibular osteotomy
2. Preemptive Kirschner wire fixation at the anticipated tibial hinge site
3. Deltoid ligament release from the medial malleolus, multiple drilling on the medial talar dome, and PTT Z-shaped cut
4. Distal syndesmotic SMO
5. Opening of the tibial osteotomy site, followed by plate fixation with a metal wedge or tricortical bone graft
6. Fibular fixation using two Kirschner wires
7. PTT repair with lengthening
8. ATFL and CFL repair or reconstruction as needed

## 6. Postoperative Management

Initially, a below-knee splint was maintained postoperatively. Once the operative wound stabilized, typically around 1 week after surgery, it was converted to a below-knee cast. During cast application, the ankle was positioned in valgus and external rotation as much as possible, and we routinely checked whether the talus was maintained in a neutral position under fluoroscopic guidance. This process facilitates the creation of space between the medial talar dome and the tibial plafond during the casting period, allowing time for the formation of fibrous cartilage at the medial talar dome. A similar principle can be applied through distraction arthroplasty to maintain the space between the medial talar dome and tibial plafond after SMO [72,73]. However, owing to concerns regarding pin tract infection and low patient compliance, we did not perform this procedure.

The cast was maintained for 6 weeks postoperatively, during which strict non-weight-bearing was essential. Patient education is crucial for ensuring adherence to this restriction. At 6 weeks postoperatively, the cast was removed, and a walking boot was applied, allowing partial weight bearing with one-crutch assistance. Full weight bearing without a brace was permitted 3 months postoperatively, with a gradual return to sports activities thereafter.

## 7. Conclusions

We have presented various currently available surgical procedures for degenerative varus ankle OA with a large talar tilt, integrating relevant literature with lessons from our clinical experience. However, surgical correction of a large varus talar tilt remains a significant challenge as no definitive solution has been established to date, necessitating further investigation and refinement.

## Figures and Tables

**Table 1:** Fundamental radiographic parameters in joint-preserving surgery for varus ankle osteoarthritis.

Parameters	Clinical Implications	Reference Range
TAS	A quantitative measure of correction	88–93°
TT	Aim of correction	≤4°
TMMA	A quantitative measure of medial malleolar erosion	20–25°
TCA	Relative fibular length measurement	8–15°
TPI	Tibial plafond coronal alignment relative to the ground (a supplementary aim of correction)	N/A
TLS	Sagittal alignment parameter of the distal fragment after correction (anterior/posterior angulation)	79–83°
HMA	A parameter assessing hindfoot alignment	Neutral alignment when the tibial bisecting line intersects the lowest point of the calcaneus
HAR	A parameter assessing hindfoot alignment	0.3–0.5

TAS, tibial anterior surface angle; TT, talar tilt; TMMA, tibiomedial malleolar angle; TCA, talocrural angle; TPI, tibial plafond inclination; TLS, tibial lateral surface angle; HMA, hindfoot moment arm; HAR, hindfoot alignment ratio.

## Data Availability

Not applicable.
